# Appraisal of COVID-19 Vaccination Acceptance in the Romanian Pregnant Population

**DOI:** 10.3390/vaccines10060952

**Published:** 2022-06-15

**Authors:** Cosmin Citu, Veronica Daniela Chiriac, Ioana Mihaela Citu, Oana Maria Gorun, Bogdan Burlea, Felix Bratosin, Daniela-Eugenia Popescu, Adrian Ratiu, Oana Buca, Florin Gorun

**Affiliations:** 1 Department of Obstetrics and Gynecology, “Victor Babes” University of Medicine and Pharmacy Timisoara, Eftimie Murgu Square 2, 300041 Timisoara, Romania; citu.ioan@umft.ro (C.C.); popescu.daniela@umft.ro (D.-E.P.); ratiu.adrian@umft.ro (A.R.); 2 Department of Internal Medicine I, “Victor Babes” University of Medicine and Pharmacy Timisoara, Eftimie Murgu Square 2, 300041 Timisoara, Romania; citu.ioana@umft.ro; 3 Department of Obstetrics and Gynecology, Municipal Emergency Clinical Hospital Timisoara, 1-3 Alexandru Odobescu Street, 300202 Timisoara, Romania; oanabalan@hotmail.com (O.M.G.); dr.burleabogdan@gmail.com (B.B.); bucaoana6@gmail.com (O.B.); gorun.florin@umft.ro (F.G.); 4 Methodological and Infectious Diseases Research Center, Department of Infectious Diseases, “Victor Babes” University of Medicine and Pharmacy, 300041 Timisoara, Romania; felix.bratosin7@gmail.com

**Keywords:** SARS-CoV-2, COVID-19, pregnancy vaccination, vaccination acceptance

## Abstract

Widespread COVID-19 vaccination is crucial for limiting the spread of SARS-CoV-2 and minimizing the risk of novel variants arising in the general population, especially in pregnant women. According to the publicly available research data, vaccination intentions vary significantly by country, with Romania among the European countries with the lowest vaccination rates. Thus, we sought to determine the scale of acceptance of the COVID-19 vaccination campaign among pregnant women in Romania, as well as the variables affecting their choices. A cross-sectional study was conducted on pregnant women referred to the Obstetrics and Gynecology Clinic of the Timisoara Municipal Emergency Hospital in Romania, where participants were asked to complete an online survey including standardized and unstandardized questionnaires indicating their willingness to receive a COVID-19 vaccine and the reasons for their willingness. Out of the 500 women who were requested to participate, there was a total of 345 validated questionnaires, with 184 vaccinated and 161 unvaccinated pregnant women. The statistically significant determinant factors for COVID-19 vaccination acceptance were the urban area of residence (OR = 0.86), having a higher level of education (OR = 0.81), the third trimester of pregnancy (OR = 0.54), trusting the government (OR = 0.83), being a frequent traveler (OR = 0.76), fearing the severity of COVID-19 (OR = 0.68), the higher availability of COVID-19 vaccines nearby (OR = 0.87), and seeing more people getting vaccinated (OR = 0.75). As there are no increased risks associated with SARS-CoV-2 immunization in pregnant women, the variables identified in this research are crucial in determining the acceptability of COVID-19 vaccines that should be addressed in this vulnerable group to increase vaccination rates.

## 1. Introduction

Coronavirus disease 2019 (COVID-19) is continuously spreading around the globe, despite the fact that exhaustive steps have been taken, including a universal vaccination campaign against the severe acute respiratory syndrome coronavirus 2 (SARS-CoV-2) virus, which is anticipated to be the most effective preventative strategy for restricting the pandemic’s spread [1,2]. The pandemic is likely to continue to cause an important rise in mortality [3,4] and disrupt global communities and economies while generating a worldwide public health disaster that continues to spread and cause severe economic and social consequences [5,6]. The increase in incidence has prompted the adoption of new treatment methods, but with little effectiveness [7,8,9]. The development of a vaccine, in particular, was pursued as a more practical and effective means to prevent and terminate the spread of SARS-CoV-2 and its detrimental effects on healthcare systems and patients with special needs [10,11,12].

In the past year, the public’s focus has been concentrated on the improvement and adoption of a vaccine that can serve as a reliable and cost-effective preventive tool against infections and severe disease forms, and a variety of COVID-19 vaccines are either in the clinical trials phase or have been approved for emergency use in many countries [13,14]. While some laboratories and private businesses have manufactured vaccines against SARS-CoV-2 using well-known methods, others have pursued the development of genetically engineered vaccines [15]. Among the most popular ones, Pfizer/BioNTech (Reinbek, Germany), AstraZeneca (Oxford, UK), Moderna (Norwood, MA, USA), and Johnson & Johnson’s Janssen (New Brunswick, NJ, USA) COVID-19 vaccines are now approved and undergoing trials while being distributed all around the globe. Despite this, corporations were under immense pressure to accelerate the manufacturing process, which led to widespread skepticism over their effectiveness and safety [16,17,18]. However, the urgency of the situation demanded the quick implementation of global immunization procedures.

With the development of effective COVID-19 vaccines, herd immunity must be achieved by the widespread vaccination of the entire population. In addition to vaccination safety, efficacy, and cost-efficiency, public acceptability is a major factor in determining overall effectiveness [19]. Since the beginning of the vaccination campaign in late 2020, there have been more than 5 billion people fully vaccinated worldwide, accounting for a total of roughly 60% of the global population [20]. In spite of strong warnings, the acceptability of the COVID-19 vaccination among the remaining 40% differs significantly across nations and groups, with distinct sociodemographic factors [21]. Even among persons with chronic medical illnesses who are at a higher risk of negative consequences associated with SARS-CoV-2 infection, COVID-19 vaccination rates remain inadequate [22,23,24]. Despite the availability of vaccination facilities, the phrase “vaccine reluctant” is widely used to characterize those who are uncertain about or unwilling to follow the guidelines [25].

A spectrum of vaccine acceptability exists, ranging from a minority that vehemently opposes all vaccinations to a majority that is prepared to take all necessary immunizations [26]. Vaccine-hesitant individuals are a heterogeneous group with varying levels of uncertainty and worries [27,28]. This group is of particular interest to public health services, as many vaccine-hesitant individuals may be influenced to change their attitudes and behaviors if their fears are handled effectively and systemic barriers to accessing health services are removed. Individuals who are not addressed about their vaccination refusal are not likely to adjust their beliefs. In order to restore society to its pre-pandemic state, it is essential to comprehend the factors that impact vaccination acceptance among various socioeconomic groups—particularly pregnant women, with their specific vulnerabilities [29,30,31]. Therefore, the goal of this study was to investigate pregnant women’s perspectives on COVID-19 vaccination, with a particular focus on the variables behind COVID-19 vaccine acceptance, with the aim of addressing these factors to improve their acceptance.

## 2. Materials and Methods

### 2.1. Study Design and Participants

From 1 January 2022 to 1 May 2022, a cross-sectional study was conducted on pregnant outpatients at the Obstetrics and Gynecology Clinic of the Timisoara Municipal Emergency Hospital, associated with the University of Medicine and Pharmacy in Timisoara, Romania. Patients were told of the purpose and consequences of the research, and each patient signed a written informed consent form to be included in the present study. The surveys were delivered online, and data collection was performed based on complete answers received in parallel with paper records of the pregnant women followed at our clinic. All patients with a history of SARS-CoV-2 infection were excluded from the study, as well as incomplete questionnaires. Our research was conducted in accordance with the Helsinki Declaration’s guidelines for scientific studies involving human participants, and it was authorized by the Scientific Ethics Committee of the Timisoara Municipal Hospital on 23 December 2021 (code I-32467/23.12.2021).

### 2.2. Surveys and Variables

A convenience sampling strategy to calculate the appropriate sample size was employed, which was estimated to comprise at least 385 pregnant women, with a margin of error of 5% at a confidence level of 95%, and a vaccination rate estimate of 50% at the time of the research [32]. Out of the 500 women who were requested to participate, 412 consented to participate in the research and fill out our questionnaires, of whom 67 failed to provide consistent and complete answers, leaving a total of 345 validated questionnaires. Out of the remaining pregnant women included in the study, 184 were vaccinated, and 161 were unvaccinated.

There were three standardized questionnaires that were validated and translated into Romanian before being given to the participants, including the HADS (Hospital Anxiety and Depression Scale), SF-12 (Short-Form Health Survey), and CORE-OM (CORE Outcome Measure Questionnaire). The HADS test is a 14-item [33] tool used to assess depression and anxiety, including 7 questions designed to measure sadness and 7 questions designed to measure anxiety. Increased scores suggest a rise in anxiety and depressive symptoms, and a score of 11 or above indicates a clinical diagnosis. The SF-12 is a regularly used tool for assessing general health and health outcomes [34], and was originally used to assess health-related quality of life. The SF-12 is a 12-item physical and mental health assessment questionnaire. The summary scores for the physical and mental components were generated in line with predetermined criteria. A low score implies poor mental and physical health. The CORE-OM is a 34-item validated self-report questionnaire with a five-point scale ranging from “never” to “most of/always” [35]. All four aspects of women’s wellbeing, concerns and symptoms, everyday functioning, and risk/harm are documented. Mean and total scores were calculated to determine the level of global psychological discomfort. A higher score suggests improved health and less global suffering in terms of wellbeing, problems and symptoms, and everyday functioning. A high risk or harm score is indicative of increased psychological distress. Another set of unstandardized questions was created by the researchers with the aim of evaluating the perceptions of pregnant women towards accepting the COVID-19 vaccines. All surveyed questions were categorical “yes” or “no”.

### 2.3. Statistical Analysis

To perform descriptive and inferential statistics, the IBM SPSS Statistics for Windows, Version 26.0 (Armonk, NY, USA, IBM Corp), was utilized. Means and standard deviations were employed to describe continuous data, while absolute values and percentages were utilized to represent categorical variables. Student’s *t*-test was used to compare the mean values of the data examined in this investigation. In a multivariate backward stepwise logistic regression analysis, all variables with statistically significant differences between groups were included. Chi-squared and Fisher’s tests were used for proportional comparisons between the two research groups. The significance threshold was established at alpha = 0.05.

## 3. Results

### 3.1. Background and Obstetrical Characteristics

At the end of the study period, a total of 184 vaccinated and 161 unvaccinated pregnant women completed the given questionnaires, and were eligible for inclusion in the study. Table 1 describes a comparison between the two study groups in their baseline characteristics. It was observed that most of the patients were in the 25–34-year-old range (51.6% vaccinated and 47.8% unvaccinated; *p*-value = 0.772). A statistically significant difference in proportions was observed in the area of residence of participants, where 42.9% of unvaccinated participants were residing in rural areas of Romania, compared with only 31% in the vaccinated group (*p*-value = 0.022). In consequence, there was a higher frequency of low-income participants in the unvaccinated group (41.6% vs. 28.8%; *p*-value = 0.042). Other statistically significant differences were observed in the level of education of the participants, where 92 (50.0%) had higher education in the vaccinated group, compared with 50 (31.1%) in the unvaccinated group (*p*-value = 0.001). Moreover, frequent alcohol consumption was more common in the unvaccinated group (5.0% vs. 3.3%; *p*-value = 0.026). Lastly, it was observed that a majority of 163 (88.6%) were vaccinated with the BNT162b2 (Pfizer/BioNTech) mRNA vaccine.

A comparison of the obstetrical and medical history of vaccinated and unvaccinated pregnant women is described in Table 2. The majority of participants were in their first pregnancy (>50% in both groups), without noteworthy differences in gravidity and parity. A statistically significant difference was observed in the trimester of pregnancy of the studied women, where 88 (47.8%) in the third trimester were vaccinated, compared to 48 (29.8%) unvaccinated in the third trimester of pregnancy (*p*-value = 0.002). Other variables—such as pregnancy-associated complications, body mass index, history of pregnancy loss, and comorbidities—did not have significant differences in proportions between the two study groups. The most common complaints of the studied women were respiratory and cardiovascular, including asthma and high blood pressure. Patients were also screened for a history of depression, without significant differences (3.3% in the vaccinated group, compared with 3.7% in the unvaccinated group; *p*-value = 0.813).

### 3.2. Standardized and Unstandardized Questionnaires

The studied participants were asked to complete a series of standardized and unstandardized surveys. Based on the hypothesis that higher levels of anxiety and depression are associated with COVID-19, hospitals are likely to determine COVID-19 vaccination. It was observed that vaccinated pregnant women scored statistically significantly higher average HADS anxiety scores (7.3 vs. 6.2; *p*-value = 0.012), HADS depression scores (6.4 vs. 5.1; *p*-value < 0.001), and HADS total scores (12.5 vs. 10.2; *p*-value < 0.001). However, there were no significant differences in completed answers for the SF-12 and CORE-OM surveys (Table 3).

**Figure 1:**
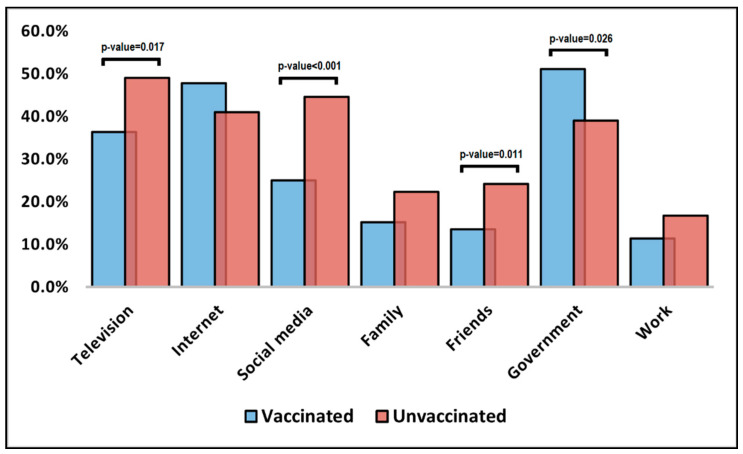
Influencing factors for COVID-19 vaccination.

**Figure 2:**
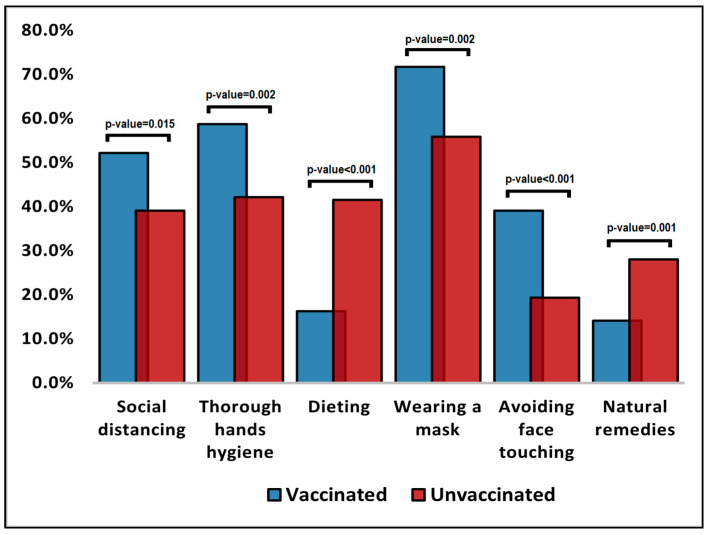
Beliefs over factors that can prevent COVID-19.

The comparison of unstandardized surveyed questions for pregnant women and their willingness to get vaccinated against COVID-19 is presented in Table 4 and in Figure 1 and Figure 2, respectively. It was observed that unvaccinated pregnant women were significantly more likely to choose television (49.1% vs. 36.4%), social media (44.7% vs. 25.0%), and friends (24.2% vs. 13.6%) as trustworthy decision factors (Figure 1). Conversely, vaccinated pregnant women were statistically significantly more likely to trust the government (51.1% vs. 39.1%; *p*-value = 0.026). The patients’ beliefs over factors that can prevent COVID-19 were significantly different in proportions between all surveyed questions (Figure 2). It was observed that vaccinated pregnant women were more likely to believe that social distancing, thorough hand hygiene, wearing a mask, and avoiding face touching are important in preventing COVID-19.

### 3.3. Factors for Acceptance

Another set of questions meant to determine COVID-19 vaccination acceptance is presented in Table 5, where patients were asked what they care about, what their concerns are, and what they believe their decision was based on. Vaccinated pregnant women were more likely to care about putting an end to the pandemic, traveling without restrictions, the severity of SARS-CoV-2 infection, COVID-19 vaccination becoming mandatory, and what doctors recommend. On the other hand, unvaccinated pregnant women were more concerned about vaccination side effects, efficacy, and the quality of vaccines delivered to Romania, while they also believed in a significantly higher proportion that COVID-19 is a conspiracy (23.0% vs. 6.0%; *p*-value < 0.001). Furthermore, the group of unvaccinated pregnant women answered that they might be influenced by the higher availability of COVID-19 vaccines nearby (42.2% vs. 22.3%; *p*-value < 0.001) and seeing more people getting vaccinated (35.4% vs. 6.5%; *p*-value < 0.001).

**Figure 3:**
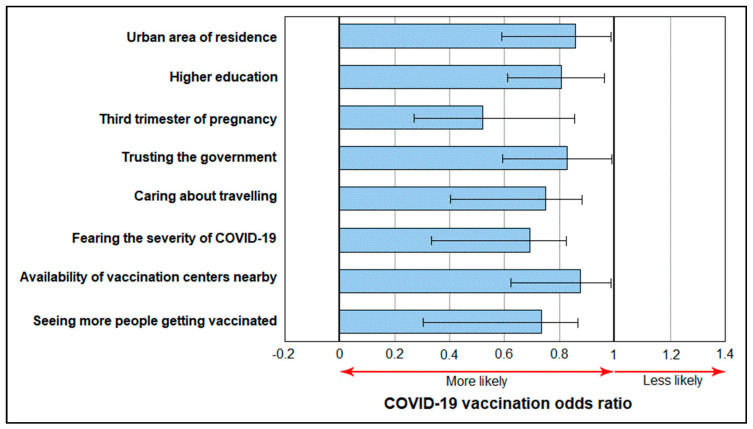
Determinant factors of the likelihood of COVID-19 vaccination.

The analysis of determinants for COVID-19 vaccine acceptance among pregnant women is described in Table 6, where a series of independent factors were observed as determining a higher likelihood of vaccination acceptance (0 > OR < 1). Among these statistically significant factors were the urban area of residence (OR = 0.86), having a higher education (OR = 0.81), the third trimester of pregnancy (OR = 0.54), trusting the government (OR = 0.83), caring about traveling (OR = 0.76), fearing the severity of COVID-19 (OR = 0.68), the higher availability of COVID-19 vaccines nearby (OR = 0.87), and seeing more people getting vaccinated (OR = 0.75), as presented in Figure 3.

## 4. Discussion

The present study managed to identify several factors that are believed to determine the acceptance of COVID-19 vaccines among pregnant women or to be associated with women who choose to vaccinate. To date, there have been several studies researching COVID-19 vaccination hesitancy and acceptance in the general population or among certain categories of people, while fewer had the purpose of studying the determining factors for acceptance among pregnant women in Romania, as a country with a COVID-19 vaccination campaign that did not develop adequately compared with the other countries in the European Union community. Therefore, as of May 2022, Malta had the highest COVID-19 immunization rate in Europe, having provided 248 doses per 100 persons, while Romania was the second-least vaccinated country in Europe, behind Bulgaria, with just 87 doses per 100 persons [36].

Although several studies reported a higher vaccination acceptance among women compared to the opposite gender, pregnant women are more reluctant due to their pregnancy status and fears with regard to the implications vaccines might have for the unborn child. As such, a recent study by Citu et al. [16] using the Vaccination Attitudes Examination (VAX) scale found that the pregnant women who completed the survey had much more reluctant responses than the non-pregnant group, with pregnant women recording significantly more hesitant responses (52% vs. 40%). They exhibited considerably higher average scores on all subscales of the VAX scale, and 78.1% attributed their COVID-19 vaccination choice to social media, compared to 64.0% of non-pregnant women. The independent risk variables for reluctance were found to be the lack of fear of COVID-19, a below-average level of income, trusting social media, a lack of belief in the existence of SARS-CoV-2, and a general lack of belief in vaccines and how they work.

Another research determined that numerous pregnant women understand the means of being safe and healthy but do not take the necessary precautions. The decision of a pregnant woman to participate in the vaccination campaign or take it seriously depends on her perceptions of the dangers caused by SARS-CoV-2 infection and the efficacy of immunization. Regardless of whether they were vulnerable, pregnant women who declined COVID-19 vaccines were observed to deny the danger of the virus or seeing any advantages from the immunizations currently in use. Acceptance was determined to be positively correlated with belief in vaccination’s advantages and self-reported patient outcomes [37].

Since pregnancy is considered to be a medical condition, pregnant women cannot participate in clinical studies of COVID-19 vaccination [38]. Thus, parents worry about the health of their unborn children due to the paucity of information about the safety and efficacy of the vaccine during pregnancy. The unknowns of the new mRNA vaccines have not been thoroughly evaluated during pregnancy in large cohorts of patients, and preliminary studies are insufficient to provide guidance for pregnant women, even though the medical advice is currently promoting vaccination [39]. Similar findings were observed in our study, where unvaccinated respondents were more likely to answer that they do not trust the efficacy of these vaccines, and that they are more concerned about the side effects.

In order to overcome these obstacles, awareness initiatives must be strengthened in order to increase the possibility of acceptance among pregnant women. It is vital to increase the perception of risk and severity among both pregnant and non-pregnant women [40]. It is vital that awareness campaigns address the concerns of pregnant women about the safety and efficacy of the vaccination during pregnancy, in order to reduce their perception of these obstacles [41]. Concerning safety, the obstetric population must be reassured that COVID-19 vaccines are not live vaccines, which are generally avoided during pregnancy because they can harm the developing fetus, and that there should be no increased reaction beyond what is anticipated [42].

The current research is restricted to a population-based analysis, so the findings of the questionnaires for pregnant women in Romania may not be relevant to other locations with different perceptions about SARS-CoV-2, as COVID-19 vaccination rates might vary significantly. Other country-specific factors that seemed to influence vaccination included rural origin, below-average income, and low levels of education, all of which are more frequent in Romania than in the rest of the EU. Other limitations include the study’s online design, as well as its failure to fulfill the estimated ideal sample size.

## 5. Conclusions

There are no increased dangers of immunization in pregnant women beyond what is anticipated. Additionally, no harmful maternal or fetal abnormalities were seen after the vaccination of pregnant women with COVID-19 vaccines. The vaccines now available are equally effective for pregnant and non-pregnant women. The claims that SARS-CoV-2 vaccines may cause infertility or impair embryonic development are unfounded, and there is no need for pregnant women to be concerned about their safety. Therefore, the factors identified in this study as important for determining the acceptance of COVID-19 vaccination should be addressed in this sensitive population to improve vaccination rates.

## Figures and Tables

**Table 1:** Comparison in baseline characteristics between vaccinated and unvaccinated pregnant women.

Variables *	Vaccinated (*n* = 184)	Unvaccinated (*n* = 161)	*p*
Age range (years)			0.772
<25	38 (20.7%)	35 (21.7%)	
25–34	95 (51.6%)	77 (47.8%)	
>34	51 (27.7%)	49 (30.4%)	
**Area of Residence**			0.022
Rural	57 (31.0%)	69 (42.9%)	
Urban	127 (69.0%)	92 (57.1%)	
**Relationship Status**			0.182
Married/concubinage	171 (92.9%)	143 (88.8%)	
Single/divorced/widowed	13 (7.1%)	18 (11.2%)	
**Income**			0.042
Low	53 (28.8%)	67 (41.6%)	
Medium	98 (53.3%)	72 (44.7%)	
High	33 (17.9%)	22 (13.7%)	
**Education**			0.001
Primary education	10 (5.4%)	16 (9.9%)	
High school	82 (44.6%)	95 (59.0%)	
Higher education	92 (50.0%)	50 (31.1%)	
**Occupation**			0.740
Employed/self-employed	153 (83.2%)	136 (84.5%)	
Unemployed	31 (16.8%)	25 (15.5%)	
**Behavior**			0.026
Frequent alcohol consumption	6 (3.3%)	8 (5.0%)	
Frequent smoker	18 (9.8%)	29 (18.0%)	
**COVID-19 vaccine**			
BNT162b2	163 (88.6%)	-	-
mRNA-1273	18 (9.8%)	-	-
Ad26.COV2.S	3 (1.6%)	-	-

* Data reported as *n* (frequency) and calculated using the chi-squared test and Fisher’s exact test unless otherwise specified; BNT162b2—Pfizer/BioNTech; mRNA-1273—Moderna; Ad26.COV2.S—Johnson & Johnson.

**Table 2:** Comparison of obstetrical and medical history between vaccinated and unvaccinated pregnant women.

Variables *	Vaccinated (*n* = 184)	Unvaccinated (*n* = 161)	*p*
**Gravidity**			0.754
1	94 (51.1%)	88 (54.7%)	
2	72 (39.1%)	60 (37.3%)	
≥3	18 (9.8%)	13 (8.1%)	
**Parity**			0.881
1	99 (53.8%)	90 (55.9%)	
2	74 (40.2%)	63 (39.1%)	
≥3	11 (6.0%)	8 (5.0%)	
**Trimester of pregnancy**			0.002
1	32 (17.4%)	40 (24.8%)	
2	64 (34.8%)	73 (45.3%)	
**3**	88 (47.8%)	48 (29.8%)	
Pregnancy-associated complications **			0.362
0	147 (79.9%)	138 (85.7%)	
1	32 (17.4%)	20 (12.4%)	
≥ 2	5 (2.7%)	3 (1.9%)	
Body mass index ***			0.780
Normal weight	136 (73.9%)	124 (77.0%)	
Overweight	30 (16.3%)	24 (14.9%)	
Obese	18 (9.8%)	13 (8.1%)	
**History of pregnancy loss**			0.371
None	146 (79.3%)	135 (83.9%)	
Medical abortion	6 (3.3%)	8 (5.0%)	
Stillbirth (<20 weeks)	13 (7.1%)	7 (4.3%)	
Miscarriage (>20 weeks)	19 (10.3%)	11 (6.8%)	
**Comorbidities**			
Cardiovascular	7 (3.8%)	4 (2.5%)	0.486
Metabolic	4 (2.2%)	4 (2.5%)	0.848
Autoimmune	3 (1.6%)	1 (0.6%)	0.382
Respiratory	9 (4.9%)	7 (4.3%)	0.810
Other	4 (2.2%)	2 (1.2%)	0.508
History of depression	6 (3.3%)	6 (3.7%)	0.813

* Data reported as *n* (frequency) and calculated using the chi-squared test and Fisher’s exact test unless otherwise specified. ** Including high blood pressure, gestational diabetes, infections, and preeclampsia. *** Adjusted for the month of pregnancy.

**Table 3:** Comparison of standardized questionnaires between vaccinated and unvaccinated pregnant women.

Variables *	Vaccinated (*n* = 184)	Unvaccinated (*n* = 161)	*p*
**HADS**			
Anxiety	7.3 ± 4.1	6.2 ± 4.0	0.012
Depression	6.4 ± 3.5	5.1 ± 2.9	<0.001
Total score	12.5 ± 5.4	10.2 ± 4.6	<0.001
**SF-12**			
Physical	56.3 ± 7.5	55.1 ± 7.0	0.127
Mental	53.8 ± 9.1	52.4 ± 8.8	0.148
Total score	55.2 ± 8.3	53.7 ± 7.6	0.082
**CORE-OM**			
CORE-W	0.89 ± 0.58	0.93 ± 0.62	0.536
CORE-P	0.71 ± 0.57	0.77 ± 0.58	0.334
CORE-F	0.63 ± 0.53	0.71 ± 0.56	0.174
CORE-R	0.26 ± 0.14	0.28 ± 0.11	0.145
Total score	0.80 ± 0.52	0.84 ± 0.55	0.488

* Data reported as *n* (frequency) and calculated using the chi-squared test and Fisher’s exact test unless otherwise specified; HADS—Hospital Anxiety and Depression Scale; SF-12—Short-Form Health Survey; CORE-OM—CORE Outcome Measure Questionnaire.

**Table 4:** Comparison of unstandardized questionnaires between vaccinated and unvaccinated pregnant women.

Variables *	Vaccinated (*n* = 184)	Unvaccinated (*n* = 161)	*p*
**Influencing factors**			
Television	67 (36.4%)	79 (49.1%)	0.017
Internet	88 (47.8%)	66 (41.0%)	0.202
Social media	46 (25.0%)	72 (44.7%)	<0.001
Family	28 (15.2%)	36 (22.4%)	0.088
Friends	25 (13.6%)	39 (24.2%)	0.011
Government	94 (51.1%)	63 (39.1%)	0.026
Work	21 (11.4%)	27 (16.8%)	0.151
**Beliefs over factors that can prevent COVID-19**			
Social distancing	96 (52.2%)	63 (39.1%)	0.015
Thorough hand hygiene	108 (58.7%)	68 (42.2%)	0.002
Dieting	30 (16.3%)	67 (41.6%)	<0.001
Wearing a mask	132 (71.7%)	90 (55.9%)	0.002
Avoiding face touching	72 (39.1%)	31 (19.3%)	<0.001
Natural remedies	26 (14.1%)	45 (28.0%)	0.001

* Data reported as *n* (frequency) and calculated using the chi-squared test and Fisher’s exact test unless otherwise specified.

**Table 5:** Assessment of reasons for COVID-19 vaccine acceptance.

Questions	Vaccinated (*n* = 184)	Unvaccinated (*n* = 161)	*p*
**“I care about”**			
Putting an end to the pandemic	166 (90.2%)	129 (80.1%)	0.007
Allowing life to return to normal	124 (67.4%)	97 (60.2%)	0.167
Travelling without restrictions	109 (59.2%)	62 (38.5%)	<0.001
The severity and complications of COVID-19	127 (69.0%)	56 (34.8%)	<0.001
COVID-19 vaccines becoming mandatory	88 (47.8%)	50 (31.1%)	0.001
What doctors recommend	70 (38.0%)	44 (27.3%)	0.034
**“I am concerned about”**			
Vaccination side effects	51 (27.7%)	84 (52.2%)	<0.001
Vaccination efficacy	48 (26.1%)	74 (46.0%)	<0.001
COVID-19 being a conspiracy	11 (6.0%)	37 (23.0%)	<0.001
Vaccination being against my religion	8 (4.3%)	12 (7.5%)	0.218
The quality of vaccines received by my country	15 (8.2%)	31 (19.3%)	0.002
Vaccine efficacy against new SARS-CoV-2 strains	19 (10.3%)	24 (14.9%)	0.198
The technology of COVID-19 vaccines	60 (32.6%)	87 (54.0%)	
**“I might be influenced by”**			
Availability of vaccines near me	41 (22.3%)	68 (42.2%)	<0.001
Seeing better results against new SARS-CoV-2 infections	45 (24.5%)	53 (32.9%)	0.082
Seeing more people getting vaccinated	26 (14.1%)	57 (35.4%)	<0.001
Clinical trials’ results	12 (6.5%)	9 (5.6%)	0.718

**Table 6:** Analysis of determinants for COVID-19 vaccine acceptance among pregnant women.

Determinants	OR *	95% CI	*p*-Value
Urban area of residence	0.86	0.59–0.98	0.043
Higher education	0.81	0.62–0.95	0.030
Third trimester of pregnancy	0.54	0.28–0.86	<0.001
Trusting the government	0.83	0.59–0.99	0.047
Caring about travelling	0.76	0.40–0.87	0.005
Fearing the severity of COVID-19	0.68	0.34–0.82	0.001
Availability of vaccination centers nearby	0.87	0.63–0.99	0.045
Seeing more people getting vaccinated	0.75	0.33–0.88	<0.001

* OR—odds ratio (a value between 0 and 1 indicating a protective effect).

## Data Availability

The data presented in this study are available upon request from the corresponding author.
